# Commensal protists in reptiles display flexible host range and adaptation to ectothermic hosts

**DOI:** 10.1128/mbio.02273-23

**Published:** 2023-11-14

**Authors:** Elias R. Gerrick, Leila B. DeSchepper, Claire M. Mechler, Lydia-Marie Joubert, Freeland Dunker, Timothy J. Colston, Michael R. Howitt

**Affiliations:** 1 Department of Pathology, Stanford University School of Medicine, Stanford, California, USA; 2 Cell Sciences Imaging Facility (CSIF), Stanford University, Stanford, California, USA; 3 Steinhart Aquarium, California Academy of Sciences, San Francisco, California, USA; 4 Biology Department, University of Puerto Rico, Mayagüez, Puerto Rico; 5 Department of Microbiology, Stanford University School of Medicine, Stanford, California, USA; 6 Program in Immunology, Stanford University School of Medicine, Stanford, California, USA; University of California Los Angeles, Los Angeles, California, USA

**Keywords:** protists, gut microbiota, microbiome-host interactions, reptiles, eukaryome, parabasalids

## Abstract

The eukaryome of mammals contains parabasalid protists that dramatically affect host immune function and health. However, the prevalence and diversity of parabasalids in wild reptiles and the consequences of captivity on these symbiotic protists are unknown. Reptiles are ectothermic, which expose their microbiomes to temperature fluctuations such as those driven by climate change. Thus, conservation efforts for threatened reptile species may benefit from understanding how shifts in temperature and captive breeding influence the microbiota, including parabasalids, to impact host fitness and disease susceptibility. Here, we surveyed intestinal parabasalids in wild reptiles from across three continents and compared these to captive animals. Reptiles harbor surprisingly few species of parabasalids compared to mammals, but these protists exhibited a flexible host range, suggesting specific adaptations to reptilian social structures and microbiota transmission. Furthermore, reptile-associated parabasalids are adapted to wide temperature ranges and survive colder temperatures significantly better than human-associated parabasalids. Colder temperatures altered the protist transcriptomes, causing increased expression of genes associated with detrimental interactions with their hosts. Our findings establish that parabasalids are widely distributed in the microbiota of both wild and captive reptiles and highlight how these protists respond to temperature fluctuations encountered in their ectothermic hosts.

**IMPORTANCE:**

Environmental factors like climate change and captive breeding can impact the gut microbiota and host health. Therefore, conservation efforts for threatened species may benefit from understanding how these factors influence animal microbiomes. Parabasalid protists are members of the mammalian microbiota that can modulate the immune system and impact susceptibility to infections. However, little is known about parabasalids in reptiles. Here, we profile reptile-associated parabasalids in wild and captive reptiles and find that captivity has minimal impact on parabasalid prevalence or diversity. However, because reptiles are cold-blooded (ectothermic), their microbiotas experience wider temperature fluctuation than microbes in warm-blooded animals. To investigate whether extreme weather patterns affect parabasalid-host interactions, we analyzed the gene expression in reptile-associated parabasalids and found that temperature differences significantly alter genes associated with host health. These results expand our understanding of parabasalids in this vulnerable vertebrate group and highlight important factors to be taken into consideration for conservation efforts.

## OBSERVATION

The intestinal microbiota critically shapes host health through its wide-ranging influence on physiology. Mammals acquire microbes from the environment, often reflecting kinship, social structure, and diet. However, reptiles frequently perform limited or no parental care and tend to have less developed social structures than many mammals (1). Furthermore, reptiles are ectothermic, and their microbiome is highly susceptible to reconfiguration by environmental temperature changes (1, 2). Climate change has an outsized negative impact on reptile species density and diversity, and the microbiomes of these ectotherms are exposed to fluctuating global temperatures (3, 4). Conservation efforts involving captive breeding programs may compound these issues, as captivity strongly impacts reptile microbiome diversity and composition (5–7). Therefore, understanding reptile microbiomes is critical for developing conservation strategies for endangered or at-risk species.

Temperature and captivity affect bacteria in reptile microbiomes (8), but their influence on symbiotic eukaryotes remains unknown (6). Protists were long ignored as part of the microbiome, but recent work highlighted their profound effects on host health (9–12). In particular, symbiotic protists in the phylum *Parabasalia* are found widely in animals from insects to humans and contribute to nutrient acquisition (13), immunomodulation (9, 10), and protection from infections (10, 11). Although a few studies identified parabasalids in reptiles, the distribution and biology of these protists are poorly understood (14–16). In this work, we cataloged the parabasalid species in the gut microbiomes of a diverse array of captive and wild reptiles. We then directly interrogated the effect of temperature on these protists and found that reptile-associated parabasalids, unlike human-associated parabasalids, adapt to temperature fluctuations by inducing massive transcriptomic remodeling with predicted effects on microbial-host interactions.

### Results

**Fig 1:**
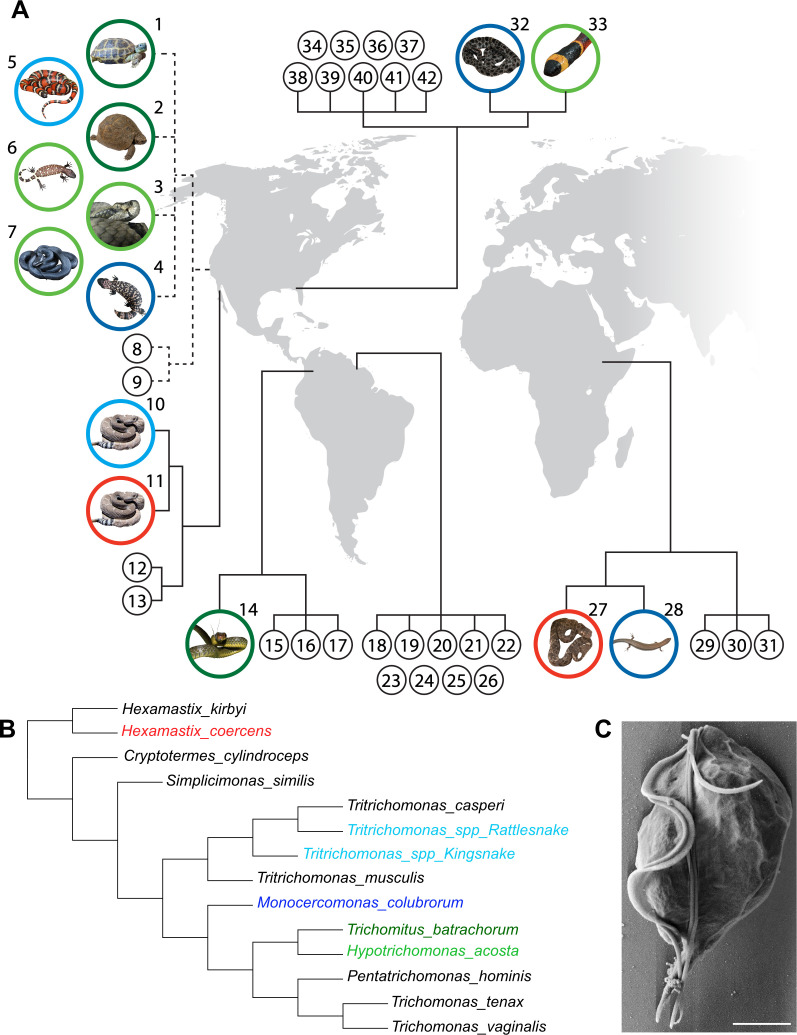
Parabasalids display limited diversity and flexible host ranges in wild and captive reptiles. (**A**) Locations and associated parabasalid protists of reptiles profiled in this study. Black numbered circles represent reptiles for which no parabasalid was detected. Colored circles indicate reptiles for which a parabasalid was identified in the sample; the image inside the circle depicts the reptile species, and the circle color indicates the parabasalid species identified in the reptile. Green circles indicate class *Hypotrichomonadea* (dark green, *Trichomitus batrachorum*; light green, *Hypotrichomonas acosta*). Blue circles indicate class *Tritrichomona*dea (dark blue, *Monocercomonas colubrorum*; light blue, *Tritrichomonas spp*.). Red circles indicate *Hexamastix coercens*. The region where each reptile was sampled is indicated on the global map (modified from Wikipedia Commons). Dashed lines denote captive reptiles, and solid lines denote wild reptiles sampled in their native habitat. (**B**) Cladogram of parabasalids identified in this study, as well as additional parabasalids of other animals including rodents and humans. Parabasalids identified in reptile samples are color-coded to match the colors in Fig. 1A. The two novel *Tritrichomonas* species identified in rodent-eating snakes are labeled with the snake in which they were identified. (**C**) Scanning electron microscopy image of *T. batrachorum* isolated from the stool of a captive *Testudo horsefieldii* tortoise. Scale bar is 2 µm.

To better characterize the diversity and prevalence of parabasalids in reptiles, we collected cloacal swabs from 33 wild reptiles across 3 continents (Fig. 1A; Table 1). In addition, we collected stool samples from nine captive reptiles to investigate the effects of captivity on parabasalid colonization and diversity. In total, this cohort represents 38 different reptile species. We designed pan-parabasalid primers to amplify the ribosomal internally transcribed spacer (ITS) to identify and phylogenetically align commensal parabasalids from these samples. Wild reptile cloacal swabs were 21% positive for parabasalids (7/33), and 78% (7/9) of the captive reptile stool samples were positive (Fig. 1A). The higher proportion of positivity in captive reptiles likely reflects the difference in sampling method, as cloacal swabs provide substantially less biomass and, thus, are more likely to produce false negatives.

**TABLE 1:** Species, parabasalid colonization status, and geographic location of captive and wild reptiles sampled

Reptile number	Reptile species	Common name	Trichomonad present	Stool/cloacal swab	Wild/captive	Reptile location
1	*Testudo horsfieldii*	Russian tortoise	*Trichomitus batrachorum*	Stool	Captive	California, USA
2	*Testudo graeca*	Greek tortoise	*Trichomitus batrachorum*	Stool	Captive	California, USA
3	*Crotalus horridus*	Timber rattlesnake	*Hypotrichomonas acosta*	Stool	Captive	California, USA
4	*Heloderma suspectum*	Gila monster	*Monocercomonas colubrorum*	Stool	Captive	California, USA
5	*Lampropeltis zonata*	Mountain kingsnake	*Tritrichomonas spp*.	Stool	Captive	California, USA
6	*Heloderma horridum*	Beaded lizards	*Hypotrichomonas acosta*	Stool	Captive	California, USA
7	*Pantherophis obsoletus*	Western ratsnake	*Hypotrichomonas acosta*	Stool	Captive	California, USA
8	*Pituophis catenifer*	Gopher snake		Stool	Captive	California, USA
9	*Elgaria multicarinata*	Alligator lizard/Lizandro		Stool	Captive	California, USA
10	*Crotalus ruber*	Red rattlesnake	*Tritrichomonas spp*.	Cloacal swab	Wild	Baja, Mexico
11	*Crotalus ruber*	Red rattlesnake	*Hexamastix coercens*	Cloacal swab	Wild	Baja, Mexico
12	*Crotalus cerastes*	Sidewinder		Cloacal swab	Wild	Baja, Mexico
13	*Pituophis catenifer*	Gopher snake		Cloacal swab	Wild	Baja, Mexico
14	*Chironius monticola*	Whipsnake	*Trichomitus batrachorum*	Cloacal swab	Wild	Colombia
15	*Anolis fuscoauratus*	Slender anole		Cloacal swab	Wild	Colombia
16	*Gonatodes concinnatus*	O'Shaughnessy Gecko		Cloacal swab	Wild	Colombia
17	*Potamites ecpleopus*	Common stream lizard		Cloacal swab	Wild	Colombia
18	*Paleosuchus trigonatus*	Smooth-fronted caiman		Cloacal swab	Wild	Guyana
19	*Platemys planicephala*	Twist-necked turtle		Cloacal swab	Wild	Guyana
20	*Chironius scurrulus*	Smooth machete savane		Cloacal swab	Wild	Guyana
21	*Corallus caninus*	Emerald tree boa		Cloacal swab	Wild	Guyana
22	*Corallus hortulanus*	Amazon tree boa		Cloacal swab	Wild	Guyana
23	*Uranoscodon superciliosus*	Diving lizard		Cloacal swab	Wild	Guyana
24	*Immantudes temnuissimus*	Yucatan blunthead snake		Cloacal swab	Wild	Guyana
25	*Bothrops asper*	Lancehead pit viper		Cloacal swab	Wild	Guyana
26	*Amphibaena fuliginosa*	Speckled worm lizard		Cloacal swab	Wild	Guyana
27	*Echis pyramidum*	Carpet viper	*Hypotrichomonas acosta*	Cloacal swab	Wild	Ethiopia
28	*Panaspis sp. nov.*	Lidless skink	*Monocercomonas colubrorum*	Cloacal swab	Wild	Ethiopia
29	*Chamaeleo dilepis*	Flap-necked chameleon		Cloacal swab	Wild	Ethiopia
30	*Hemidactylus isolepis*	Scaly leaf-toed gecko		Cloacal swab	Wild	Ethiopia
31	*Lygodactylus keniensis*	Parker’s dwarf gecko		Cloacal swab	Wild	Ethiopia
32	*Sitrurus miliarius*	Rough green snake	*Monocercomonas colubrorum*	Cloacal swab	Wild	Florida, USA
33	*Micrurus fulvis*	Eastern coral snake	*Hypotrichomonas acosta*	Cloacal swab	Wild	Florida, USA
34	*Pantherophis guttatus*	Corn snake		Cloacal swab	Wild	Florida, USA
35	*Thamnophis sirtalis*	Garter snake		Cloacal swab	Wild	Florida, USA
36	*Nerodia fasciata*	Banded water snake		Cloacal swab	Wild	Florida, USA
37	*Pantherophis obsoletus*	Western ratsnake		Cloacal swab	Wild	Florida, USA
38	*Ophisaurus attenuatus*	Slender glass lizard		Cloacal swab	Wild	Florida, USA
39	*Diadophis punctatus*	Ringneck snake		Cloacal swab	Wild	Florida, USA
40	*Coluber constrictor*	Eastern racer		Cloacal swab	Wild	Florida, USA
41	*Crotalus adamanteus*	Eastern diamondback rattlesnake		Cloacal swab	Wild	Florida, USA
42	*Opheodrys aestivus*	Rough green snake		Cloacal swab	Wild	Florida, USA

We identified six parabasalid species in these samples, but only four of these species (*Hypotrichomonas acosta*, *Trichomitus batrachorum*, *Monocercomonas colubrorum*, and *Hexamastix coercens*) have been previously found in reptiles. The other two protists were novel species of the genus *Tritrichomonas* and phylogenetically clustered with the murine-associated protists *Tritrichomonas musculis* and *Tritrichomonas casperi* (Fig. 1B). Because these novel *Tritrichomonas* species are closely related to *T. casperi* and *T. musculis* (17), we hypothesize that protist DNA from mice consumed by the captive snakes may have generated these ITS sequences. Of the remaining four parabasalid species identified in the wild reptiles, all but *H. coercens* were found in at least one captive reptile. To confirm that the reptile-associated parabasalid DNA sequences represent resident microbes, we isolated protists from the fresh stool of a domestic tortoise (*Testudo horsfieldii*) and identified motile *T. batrachorum* trophozoites that matched the ITS sequence from the unpurified stool DNA sample (Fig. 1C). Altogether, the substantial overlap between parabasalid species in wild and captive reptiles and the high frequency of colonization in captive reptiles suggests that captivity does not have a detrimental impact on either parabasalid diversity or frequency in reptiles.

This survey also revealed a surprisingly low level of diversity among reptile-associated parabasalids. We identified only four reptile-associated species despite sampling broad host and geographical ranges (Fig. 1A; Table 1). The discovery of two new rodent-associated parabasalids in the intestines of rodent-eating snakes but no new reptile-associated protists was particularly striking. These data suggest that reptile-associated parabasalids have lower diversity than their mammal-associated counterparts. Supporting this hypothesis was the presence of *T. batrachorum* in both the captive tortoises (*Testudo horsfieldii* and *Testudo graeca*) in California and the wild whipsnake (*Chironius monticola*) in Colombia. These tortoises are strict herbivores, whereas whipsnakes are strict carnivores, and thus, *T. batrachorum* appears adapted to hosts with diverse dietary patterns. This is particularly surprising because mammal-associated intestinal parabasalids have more specialized host ranges, suggesting that colonization of reptiles may require increased flexibility. Possible explanations for this phenomenon include less complex social structures than mammals, low incidence of vivipary, and rarity of parental involvement with offspring.

**Fig 2:**
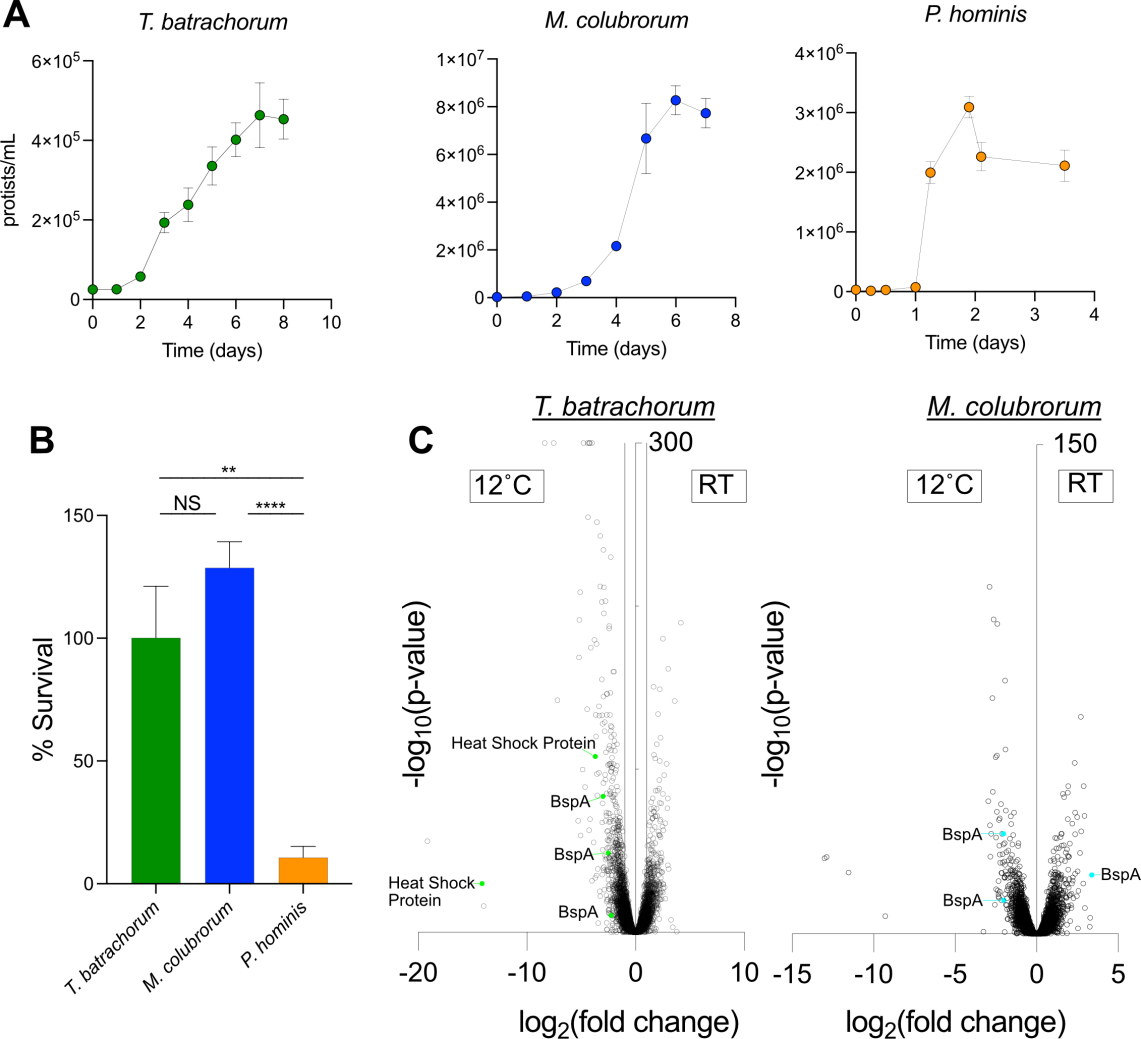
Reptile-associated parabasalids survive cold temperature and dramatically remodel their transcriptional profiles. (**A**) Growth curves of *T. batrachorum, M. colubrorum*, and *P. hominis* at their ideal growth temperatures (room temperature for reptile-associated protists or 37°C for *P. hominis*). (**B**) Survival of the three protist species during overnight culture at 12°C. (**C**) Transcriptomic changes in *T. batrachorum* (left) and *M. colubrorum* (right) during growth at 12°C. Transcripts with increased expression during cold exposure are shown on the left side of each plot. Select differentially expressed genes are colored (*T. batrachorum*, green; *M. colubrorum*, blue) and annotated with the gene function. NS, not significant; **P < 0.01, ****P < 0.0001.

Parabasalids that colonize endothermic mammals experience relatively stable temperatures, while protists in ectothermic reptiles experience substantial temperature fluctuations associated with daily and seasonal variations. In particular, parabasalids are exposed to much colder *in vivo* temperatures in reptiles than mammals. To look for common adaptations of diverse reptile-associated parabasalids, we cultured *T. batrachorum*, a member of class *Hypotrichomonadea*, and *M. colubrorum*, a member of class *Tritrichomonadea* (Fig. 2A). We compared these protists to a human-associated species, *Pentatrichomonas hominis*, which colonizes endothermic mammals (Fig. 2A). We then tested whether human- or reptile-associated species could survive cold temperature by growing these three protists at 12°C overnight. As expected, this cold exposure was lethal for *P. hominis*, but the reptile-associated species survived and even grew as motile trophozoites at 12°C (Fig. 2A and B). These data support the hypothesis that reptile-associated parabasalids have adapted to withstand substantial temperature fluctuations experienced within their ectothermic hosts.

To determine whether cold exposure may affect the interactions of *T. batrachorum* and *M. colubrorum* with their hosts, we performed RNA sequencing and *de novo* transcriptome assembly on each protist grown at either room temperature or 12°C. Cold exposure resulted in drastic transcriptional responses in both protists, as 1,808 (14.9%) and 977 (5.2%) of detected transcripts were differentially expressed between the two temperatures in *T. batrachorum* and *M. colubrorum*, respectively (Fig. 2C; Tables S1 and S2). BspA proteins are a family of surface proteins implicated in *Trichomonas vaginalis* adhesion to host epithelial cells (18). Strikingly, the three most differentially regulated *bspA* genes in *T. batrachorum* were upregulated at 12°C, while two of the three most differentially regulated *bspA* genes in *M. colubrorum* increased at cold temperatures (Fig. 2C). These findings reveal that both reptile-associated parabasalids upregulate putative host adhesion proteins in response to cold exposure. Because adhesion to the epithelium can trigger intestinal immune pathways (19), colder temperatures are predicted to drastically change protist-host interactions.

### Discussion

Parabasalid protists are increasingly recognized as part of animal microbiomes with significant influences on their hosts’ health and immune function. This study presents the first survey of these microbes in wild reptiles sampled across three continents and a diverse collection of captive reptiles. We found that parabasalid diversity is unusually low in reptiles compared to their mammalian counterparts and that reptile-associated protists are highly flexible in their host range, suggestive of adaptations to the asocial lifestyle commonly observed in reptiles. Our results also demonstrate that although captivity does not affect the diversity or prevalence of parabasalids in reptile microbiomes, the extreme weather patterns caused by climate change may dramatically affect the interactions of these microbes with their hosts. Specifically, *T. batrachorum* and *M. colubrorum* upregulate potentially detrimental genes during cold exposure, suggesting that extreme weather conditions may force commensal parabasalids to adopt pathobiont properties. Because the microbiota, including microeukaryotes like protists, critically impacts animal health, conservation efforts will be aided by understanding how captivity and environmental factors like climate change shape the composition and interactions of these microbes with vulnerable animal species.

### Materials and methods

#### Cloacal swabs and stool collection

Cloacal swabs have been shown to be an effective proxy for collecting the diversity of bacteria present in the gastrointestinal tract of reptiles (20, 21). Swabs were collected using established methods for sampling reptile cloacal microbiomes (20, 22). Animals were handled under approved IACUC protocols (U of Mississippi SOP13-04; Florida State University #1836). Briefly, animals were either captured by hand or via pitfall traps according to approved protocols during biodiversity survey expeditions undertaken by T. J. Colston from 2010 to 2020. Once restrained, the exterior of the cloaca was cleaned with alcohol swabs or 95% ethanol, then a sterile nylon tipped swab was inserted into the cloaca and rotated 10 times, taking care not to penetrate into the large intestine. Swabs were then either placed in individual empty 1.5 mL cryovials and immediately frozen in liquid nitrogen (20) or placed in 1.5 mL cryovials containing 750 µL Xpedition RNA/DNA Shield (Zymo Research Products) and stored at ambient temperature before transportation to the laboratory where they were subsequently stored at −20°C until DNA extraction (23). For captive reptiles, fresh stool samples were collected and frozen immediately.

#### DNA extraction and PCR

DNA was extracted from cloacal swab and stool samples using the Powersoil Pro DNA Extraction kit (Qiagen) according to the manufacturer’s instructions. Samples were screened for the presence of parabasalid protists by amplifying DNA using custom pan-parabasalid primers, which we designed to bind to regions of the ITS that are highly conserved among the parabasalid lineage (panParabasalid-F 5′-CCACGGGTAGCAGCA-3′ and panParabasalid-R 5′-GGCAGGGACGTATTCAA-3′). These primers amplify an approximately 1.1 kb ITS amplicon. DNA was amplified using the Accustart II Geltrack Supermix (Quantabio) with the use of touchdown PCR, with a starting annealing temperature of 72°C dropping to 65°C over 15 cycles, followed by 35 cycles with an annealing temperature of 65°C. A 1-min extension at 72°C was used for both stages. The presence of parabasalid DNA in the samples was initially assessed by agarose gel confirmation, followed by verification and species identification using Sanger Sequencing (Molecular Cloning Lab). Phylogenetic trees were created using MAFFT (24) based on ITS sequences, using default parameters.

#### Isolation of *T. batrachorum*

*T. batrachorum* was isolated from fresh stool of a captive *Testudo horsefieldii* tortoise. Isolation and culture were performed as described previously (17). Briefly, the stool sample was washed three times in sterile phosphate-buffered saline (PBS) and then resuspended in 40% percoll. This slurry was then underlaid with 80% percoll and centrifuged at 1,000 *g* for 10 min with no brake. The interphase, containing *T. batrachorum*, was washed twice with sterile PBS and then resuspended in growth medium. Protists were then grown in an anaerobic chamber at room temperature (Coy Laboratory Products). *M. colubrorum* strain Ns-1PRR and *P. hominis* strain Hs-3:NIH were obtained from American Type Culture Collection (ATCC). Both reptile-associated protists were grown at room temperature, whereas *P. hominis* was grown at 37°C. For growth curves and survival tests, protists were counted using a hemocytometer.

#### Scanning electron microscopy (SEM)

SEM was performed as described previously (9). Briefly, protists were adhered to poly-lysine-coated coverslips. Samples were then fixed in 2.5% glutaraldehyde in 0.1M cacodylate, pH 7.2. Samples were then rinsed and post-fixed for 30 min in 1% OsO_4_ in 0.1M cacodylate buffer and dehydrated in a graded series of ethanol prior to critical point drying with liquid CO_2_. Protists were then sputter-coated with 5 nm platinum and examined with a Zeiss Sigma scanning electron microscope.

#### Cold exposure survival assay

*P. hominis* strain Hs-3:NIH and *M. colubrorum* strain Ns-1PRR were obtained from ATCC. Protists were cultured in triplicate at their respective normal growth temperatures until they reached mid-log phase. Each culture was then divided in two, with one culture continuing growth at normal temperature and one being placed at 12°C. Protists were cultured overnight at the two temperatures, and then viable protists were counted in each condition on a hemocytometer.

#### RNA sequencing and *de novo* transcriptome assembly

RNA was isolated from mid-log phase protists grown at room temperature or 12°C using the Direct-zol RNA Miniprep kit (Zymo). RNA libraries were generated using the KAPA Stranded mRNA-Sequencing Kit (Roche), with poly-dT enrichment of mRNA. Samples were sequenced on a HiSeq (Illumina) using 2 × 150 read lengths. Because no genome sequences exist for *T. batrachorum* or *M. colubrorum*, *de novo* transcriptome assembly was performed using Trinity (25). Transcript abundances were obtained using kallisto (26) and differential expression analysis was performed using DESeq2 (27).

## Data Availability

Sequence data that support the findings of this study have been deposited in the Stanford Digital Repository. The transcriptomics data for *M. colubrorum* are found on SRA with BioProject accession number PRJNA1007713. The transcriptomics data for *T. batrachorum* are found on SRA with BioProject accession number PRJNA1007265.

## SUPPLEMENTAL MATERIAL

Supplemental Captions mbio.02273-23-s0001.docx Captions for Table S1 and Table S2.

Table S1 mbio.02273-23-s0002.xlsx List of differentially expressed genes in *T. batrachorum* grown at room temperature versus 12˚C.

Table S2 mbio.02273-23-s0003.xlsx List of differentially expressed genes in *M. colubrorum* grown at room temperature versus 12˚C.

ASM does not own the copyrights to Supplemental Material that may be linked to, or accessed through, an article. The authors have granted ASM a non-exclusive, world-wide license to publish the Supplemental Material files. Please contact the corresponding author directly for reuse.
