# Supplementary material for: Commensal protists in reptiles display flexible host range and adaptation to ectothermic hosts
Source: mBio. 2023 Nov 14;14(6):e02273-23. doi: 10.1128/mbio.02273-23 (PMC10746265; doi:10.1128/mbio.02273-23)
Supplement: Supplemental Captions — Captions for Table S1 and Table S2. [file mbio.02273-23-s0001.docx]

**Supplemental Figure Legends**

**Table S1: List of differentially expressed genes in *T. batrachorum* grown at room temperature vs. 12˚C.**

**Table S2: List of differentially expressed genes in *M. colubrorum* grown at room temperature vs. 12˚C.**
